# An evaluation of coronary artery plaque burden in asymptomatic type 2 diabetics using dual-source CT coronary angiography

**DOI:** 10.1186/1753-6561-6-S4-O20

**Published:** 2012-07-09

**Authors:** M Patel, VS Mehta, S Venuraju, A Yerramasu, A Jeevarethinam, S Atwal, A Lahiri

**Affiliations:** 1 University College London Medical School, UK; 2 Clinical Imaging and Research Centre Wellington Hospital, London, UK; 3 Imperial College, London, UK; 4 Middlesex University, London, UK

## Introduction

Current International Diabetes Federation (IDF) and American Diabetes Association (ADA) guidelines advocate investigation for coronary artery disease (CAD) in only those patients who have either typical/atypical cardiac symptoms or an abnormal resting ECG. As it is well established that diabetics with CAD may be asymptomatic, we hypothesised that a large proportion of asymptomatic diabetics may have coronary arterial plaques causing significant stenosis which goes undetected with current guidelines.

## Methods

A cohort of 71 type 2 diabetic patients with no known CAD was selected. Patients with coronary stents or CABG surgery were excluded. Information regarding risk factors for CAD was collected for all patients. All patients in the study provided written consent. Mean age of the cohort was 60.3 ± 8.2, with 78.85% being male. All patients underwent a CT coronary angiogram (CTCA). CTCA was reconstructed at the best diastolic phase, and plaques were categorised as non-significant (causing <50% stenosis) or significant (causing >50% or 70% stenosis). Categorical variables were presented as percentages and continuous variables as mean ± standard deviation.

## Results

We found only 16.9% of the cohort had normal coronary arteries, 80.2% had at least one plaque causing non-significant stenosis (1-49%). 56.3% of the cohort had at least one plaque causing stenosis of >50%. 33.8% patients had at least one plaque causing stenosis of >70%. We also found that this cohort had per patient, 1.17± 1.9 non-calcified plaques (NCP), 3.06± 3.33 calcified plaques, and 1.06± 1.4 mixed plaques (MP).

## Conclusions

Our study demonstrates that more than half of asymptomatic diabetic patients have at least one plaque causing stenosis of >50% and more than a third have at least one plaque causing stenosis of >70%. This suggests a revision of guidelines is needed for CAD screening in type 2 diabetic patients in order to reduce mortality and morbidity. A limitation of our study is that we have not taken into account duration of diabetes and control of blood sugar levels during this time.

